# Measuring treatment impacts on symptoms in adults with hypoparathyroidism: findings from the PaTHway trial

**DOI:** 10.1186/s41687-024-00757-1

**Published:** 2024-08-13

**Authors:** Meryl Brod, Kathryn M. Pfeiffer, Jane F. Beck, Alden Smith

**Affiliations:** 1 The Brod Group, 219 Julia Ave., Mill Valley, CA 94941 USA; 2 Ascendis Pharma, Inc., 1000 Page Mill Road, Palo Alto, CA 94304 USA

**Keywords:** Hypoparathyroidism, Adults, Exit interviews, Patient-reported outcomes, Symptoms

## Abstract

**Background:**

Hypoparathyroidism is a rare endocrine disease frequently associated with serious physical and cognitive symptoms. This study’s purpose was to understand the impacts of the phase 3 PaTHway clinical trial treatment, TransCon PTH, on patients’ overall, physical, and cognitive hypoparathyroidism signs/symptoms and what patients consider meaningful improvement.

**Methods:**

Individual telephone exit interviews were conducted with patients who recently completed the PaTHway trial blinded period. Using a semi-structured interview guide, interviews focused on trial treatment impact on hypoparathyroidism symptoms following the symptom list in the Hypoparathyroidism Patient Experience Scale-Symptom (HPES-Symptom). Meaningful changes in hypoparathyroidism symptoms were assessed with the Patient Global Impression of Severity (PGIS) and Patient Global Impression of Change (PGIC) measures. Interviewees were probed on the meaningfulness of reported changes in symptoms from prior to starting trial treatment to the past 2 weeks/current time. Interviews were audiotaped and transcribed. Transcripts were coded for emerging concepts and themes/subthemes covered in the interview guide based on an adapted grounded theory approach.

**Results:**

Nineteen adults with hypoparathyroidism participated in interviews in the United States (*n* = 13, 68.4%) and Canada (*n* = 6, 31.6%). Marked improvements in physical and cognitive symptoms were described among trial treatment group respondents. The majority of participants who reported experiencing hypoparathyroidism physical symptoms pre-trial indicated symptom improvement with treatment, including muscle twitching (100%, *n* = 15), low energy (92.9%, *n* = 13), feeling tired (92.3%, *n* = 12), muscle weakness (92.9%, *n* = 13), tingling without numbness (84.6%, *n* = 11), trouble sleeping (92.3%, *n* = 12), muscle cramping (92.3%, *n* = 12), tingling with numbness (92.3%, *n* = 12), muscle spasms (100%, *n* = 12), and pain (90.9%, *n* = 10). Most participants who reported experiencing cognitive symptoms pre-trial reported symptom improvement with treatment, including difficulty finding the right words (86.7%, *n* = 13), difficulty concentrating (93.3%, *n* = 14), trouble remembering (92.9%, *n* = 13), trouble thinking clearly (85.7%, *n* = 12), and difficulty understanding information (83.3%, *n* = 10). Those in the placebo group reported limited or no improvement. The vast majority of participants affirmed that the improvements they experienced in symptom frequency on the PGIS/PGIC and HPES–Symptom were meaningful.

**Conclusions:**

Findings indicate that TransCon PTH treatment improved participants’ physical and cognitive hypoparathyroidism symptoms in meaningful ways, while reducing the daily burden associated with conventional therapy.

**Trial registration:**

NCT04701203 Registered: 06 January 2021. https://clinicaltrials.gov/study/NCT04701203?term=NCT04701203&rank=1.

## Background

Hypoparathyroidism is an endocrine disease caused by insufficient levels of parathyroid hormone (PTH) which may lead to hypocalcemia (low blood calcium levels), hyperphosphatemia (elevated blood phosphate levels), or overly-mineralized bone [1–3]. Hypoparathyroidism most often occurs following neck surgery (approximately 75% of cases), but may also be genetic, autoimmune, or idiopathic in origin [1–3]. Hypoparathyroidism is frequently associated with serious physical symptoms, including neuromuscular issues (e.g., muscle cramping/spasms, tingling/prickling sensation, seizures), fatigue, sleep disturbance, and cognitive symptoms, e.g., inability to concentrate/focus [2].

Oral calcium and/or active vitamin D are the current conventional therapy for hypoparathyroidism. For adults who respond unsatisfactorily to conventional therapy, PTH replacement therapy may also be prescribed. Research has shown that even with conventional therapy, patients with hypoparathyroidism continue to experience physical and cognitive symptoms and a reduced quality of life (QOL) [4]. More effective treatment is needed to treat hypoparathyroidism patients’ physical and cognitive symptoms adequately and to improve QOL.

TransCon PTH (palopegteriparatide) is an investigational prodrug of PTH (1–34), administered once daily, with sustained release of active PTH designed to provide PTH levels in the physiological range for 24 h/day in adults with hypoparathyroidism. TransCon PTH is being tested in the phase 3 PaTHway clinical trial. The trial is a multicenter, randomized, double-blind, placebo-controlled (4-week screening period, 26-week treatment period (blinded)) study, which is currently in an open-label extension of 156 weeks, designed to evaluate the safety and efficacy of TransCon PTH administered subcutaneously once daily in adults with hypoparathyroidism.

This was a qualitative exit interview study of patients who participated in the PaTHway trial and recently completed the blinded period. This study’s purpose was threefold, with the aim of investigating: (1) trial treatment impacts on patients’ overall, physical, and cognitive hypoparathyroidism signs/symptoms, (2) what patients consider meaningful improvement in their hypoparathyroidism signs/symptoms; and (3) to investigate patients’ general experiences and satisfaction with the PaTHway trial treatment and treatment preferences.

The findings will provide insights, from the patient perspective, into the impacts of TransCon PTH on physical and cognitive symptoms associated with hypoparathyroidism, as well as broader impacts on patient functioning and QOL. The results will also provide qualitative evidence to inform the interpretation of symptom changes and to better understand what constitutes meaningful change in symptoms for adults with hypoparathyroidism. The study findings will be of interest to researchers, clinicians who treat adults with hypoparathyroidism, adults diagnosed with hypoparathyroidism, and to payers.

## Methods

This is a cross-sectional, qualitative exit interview study based on a sample of adults with hypoparathyroidism who recently completed a clinical trial treatment study. Individual telephone interviews were conducted with participants recruited from 11 clinical sites in North America, including 9 in the United States (US) and 2 in Canada. To be eligible for participation in the interview study, participants must have recently completed the PaTHway trial blinded period (within 2 weeks). Clinical trial key criteria included: Inclusion—adult aged 18 years or older; having a diagnosis of hypoparathyroidism (postsurgical chronic hypoparathyroidism, or auto-immune, genetic, or idiopathic hypoparathyroidism for at least 26 weeks); resides in the US or Canada; Exclusion—Impaired responsiveness to PTH (pseudohypoparathyroidism) and any disease that might affect calcium metabolism or calcium phosphate homeostasis or PTH levels other than hypoparathyroidism. The treatment to placebo randomization ratio for the trial was 3:1.

### Measures

Hypoparathyroidism Patient Experience Scale-Symptom (HPES-Symptom) [5, 6]—This patient-reported outcome measure has been shown to have sound content validity and good psychometric properties. The measure contains 17 items to assess the frequency of hypoparathyroidism-specific symptoms experienced by adults with hypoparathyroidism over the past 2 weeks. It was developed following FDA guidelines [7] and best practices for patient-reported outcome (PRO) measure development [8–11], and included literature review, expert and patient interviews, and cognitive debriefing of items. The measure can be scored for each domain or a total score. The measure uses a 5-point response option scale ranging from “never” to “very often/always”.

The Patient Global Impression of Severity (PGIS)—Overall symptom measure captures the frequency of overall hypoparathyroidism symptoms using a 6-point response scale ranging from “always” to “never”.

The Patient Global Impression of Change (PGIC)—Change in overall symptoms measure assesses participants’ reported change in how often they experienced overall symptoms due to hypoparathyroidism currently compared to the study start using a 5-point response option scale ranging from “much more often” to “much less often”.

### Interviews

A semi-structured interview guide was developed based on a literature review and previous findings from the concept elicitation interview study of adults with hypoparathyroidism conducted by The Brod Group for the development of the HPES-Symptom. Interviews covered patients’ general trial treatment experiences, their overall experiences with the trial, perceptions of trial treatment efficacy, overall treatment satisfaction, and treatment preferences using open-ended questions and follow-up probes as needed. Interviews were conducted within two weeks of the participant completing the randomized, double-blind portion of the trial.

The interview guide also asked participants more structured questions about the frequency of their hypoparathyroidism symptoms they experienced both before and after the trial. The guide followed the symptom list included in the HPES-Symptom measure and investigated the physical and cognitive domains, as well as overall hypoparathyroidism symptoms. To assess meaningful changes in hypoparathyroidism symptoms over the course of the blinded trial period, the PGIS and PGIC questions were asked of participants. Interviewees were then probed on the meaningfulness of any reported changes in overall symptoms from prior to trial treatment start to the past 2 weeks/current time, as well as what other hypothetical changes in overall hypoparathyroidism symptoms would mean to them.

Telephone interviews lasted approximately 90 min and were audio recorded and transcribed verbatim. Treatment group assignments were blinded at the time that interviews were conducted. Treatment assignment data were shared with The Brod Group after completion of the interviews and once the trial blind had been broken.

The exit interview study was part of the PaTHway trial protocol and Institutional Review Board (IRB) review and approval. The exit interview was considered a required “visit” within the clinical trial protocol and IRB approved consent.

### Data analysis

Participant demographics and health characteristics were summarized using descriptive statistics (means, standard deviations, ranges, and frequencies/percentages). Dedoose, a mixed-method qualitative data analysis software, was used for all qualitative data analysis [12]. Interview transcripts were coded for emerging concepts and themes/subthemes covered in the interview guide based on an adapted grounded theory approach [9]. Coded concepts were revised and organized into larger themes and domains through an iterative process. The interview findings were then examined and summarized by the major symptom and trial treatment experience domains addressed in the interview guide.

## Results

### Sample description

Nineteen adults with hypoparathyroidism participated in telephone interviews in the US (*n* = 13, 68.4%) and Canada (*n* = 6, 31.6%). Participant demographic characteristics, general health and disease characteristics, and hypoparathyroidism treatment characteristics by treatment group are presented in Table 1.

**Table 1:** Participant characteristics, general health and disease characteristics, and hypoparathyroidism treatment characteristics by treatment group

	TransCon PTH (*n*=15)		Placebo (*n*=4)		Total (*n*=19)	
**Participant characteristics**						
Age (years)						
Mean (SD)	45.9	(11.9)	48.0	(12.2)	46.3	(11.6)
(Range)	(22–69)		(39–66)		(22–69)	
Sex, *n* (%)						
Female	13	(86.7)	3	(75.0)	16	(84.2)
Male	2	(13.3)	1	(25.0)	3	(15.8)
Employment status, *n* (%)						
Full-time	8	(53.3)	4	(100.0)	12	(63.2)
Part-time	3	(20.0)	0	(0)	3	(15.8)
Unemployed	2	(13.3)	0	(0)	2	(10.5)
Retired	2	(13.3)	0	(0)	2	(10.5)
Marital status, *n* (%)						
Married	9	(60.0)	3	(75.0)	12	(63.2)
Single	5	(33.3)	0	(0)	5	(26.3)
Divorced	1	(6.7)	1	(25.0)	2	(10.5)
Ethnicity, *n* (%)						
White/Caucasian	14	(93.3)	4	(100.0)	18	(94.7)
Asian & White/Caucasian	1	(6.7)	0	(0)	1	(5.3)
Education level, *n* (%)						
High school or technical school	3	(20.0)	0	(0)	3	(15.8)
Some college	3	(20.0)	2	(50.0)	5	(26.3)
College degree	5	(33.3)	0	(0)	5	(26.3)
Post-graduate/professional degree	4	(26.7)	2	(50.0)	6	(31.6)
**Participant general health and disease characteristics**						
Type of hypoparathyroidism, *n* (%)						
Post-surgical	12	(80.0)	3	(75.0)	15	(78.9)
Idiopathic	3	(20.0)	0	(0)	3	(15.8)
Genetic	0	(0)	1	(25.0)	1	(5.3)
Years with hypoparathyroidism						
Mean (SD)	9.7	(7.6)	12.3	(12.0)	10.3	(8.4)
(Range)	(1–27)		(4–30)		(1–30)	
General health (self-reported at time of interview), *n* (%)						
Excellent	1	(6.7)	0	(0)	1	(5.3)
Very Good	5	(33.3)	0	(0)	5	(26.3)
Good	6	(40.0)	2	(50.0)	8	(42.1)
Fair	3	(20.0)	2	(50.0)	5	(26.3)
Poor	0	(0)	0	(0)	0	(0)
Number of comorbidities						
Mean (SD)	3.9	(1.0)	5.5	(0.8)	4.2	(3.0)
(Range)	(0–11)		(3–8)		(0–11)	
Number of prescription medications^a^						
Mean (SD)	2.7	(1.1)	3.3	(0.3)	2.8	(1.0)
(Range)	(0–6)		(3–4)		(0–6)	
Level of difficulty managing hypoparathyroidism before trial, *n* (%)						
Extremely difficult	4	(26.7)	1	(25.0)	5	(26.3)
Very difficult	8	(53.3)	2	(50.0)	10	(52.6)
Somewhat difficult	2	(13.3)	1	(25.0)	3	(15.8)
A little difficult	1	(6.7)	0	(0)	1	(5.3)
Not at all difficult	0	(0)	0	(0)	0	(0)
Level of difficulty managing hypoparathyroidism after trial, *n* (%)						
Extremely difficult	0	(0)	1	(25.0)	1	(5.3)
Very difficult	0	(0)	1	(25.0)	1	(5.3)
Somewhat difficult	4	(26.7)	1	(25.0)	5	(26.3)
A little difficult	4	(26.7)	1	(25.0)	5	(26.3)
Not at all difficult	7	(46.7)	0	(0)	7	(36.8)
**Participant hypoparathyroidism treatment characteristics**						
Prescription/OTC medications used to treat hypoparathyroidism before trial, *n* (%)						
Oral calcium (OTC)	15	(100.0)	4	(100.0)	19	(100.0)
Active Vitamin D (prescription) (e.g., Calcitriol or alfacalcidol)	15	(100.0)	4	(100.0)	19	(100.0)
Vitamin D (OTC)	10	(66.7)	4	(100.0)	14	(73.7)
Magnesium (OTC)	10	(66.7)	3	(75.0)	13	(68.4)
Hydrochlorothiazide (HCTZ)	4	(26.7)	1	(25.0)	5	(26.3)
Natpara®	2	(13.3)	0	(0)	2	(10.5)
Forteo®	1	(6.7)	0	(0)	1	(5.3)
Potassium chloride (prescription)	1	(6.7)	0	(0)	1	(5.3)
Oral contraception	1	(6.7)	0	(0)	1	(5.3)

*HCTZ *hydrochlorothiazide, *OTC* over the counter, *SD* standard deviation

^a^For conditions other than hypoparathyroidism

### Most bothersome and most important signs/symptoms of hypoparathyroidism

#### Most bothersome hypoparathyroidism signs/symptoms pre-trial

Participants reported a wide range of hypoparathyroidism physical and cognitive signs/symptoms that were most bothersome to them before starting the trial treatment, and most noted more than one symptom (Table 2). The signs/symptoms that were most frequently indicated to be the most bothersome included feeling tired (42.1%, *n* = 8), pain (26.3%, *n* = 5), muscle twitching (15.8%, *n* = 3), muscle cramping (15.8%, *n* = 3), low energy (15.8%, *n* = 3), and trouble sleeping (15.8%, *n* = 3):

Oh, man! I would say equally, they all [symptoms] were a nightmare. I would say the sleep was a big one, the sleep was a nightmare. Because, even if I wasn’t feeling like an intense brain fog or mental, like cognitive function issues, like the following day, it would just be because of the lack of sleep then. I would say that was definitely the biggest one. But, also…the physical symptoms were very tough…it made it virtually impossible to exercise…you feel worse when you don’t exercise as it is… (22-year-old male).

**Table 2:** Hypoparathyroidism signs/symptoms most bothersome to participants pre-trial

*n* (%) reporting	TransCon PTH (*n* = 15)		Placebo (*n* = 4)		Total (*n* = 19)	
Feeling tired	6	(40.0)	2	(50.0)	8	(42.1)
Pain	3	(20.0)	2	(50.0)	5	(26.3)
Muscle twitching	3	(20.0)	0	(0.0)	3	(15.8)
Muscle cramping	2	(13.3)	1	(25.0)	3	(15.8)
Low energy	2	(13.3)	1	(25.0)	3	(15.8)
Trouble sleeping	3	(20.0)	0	(0.0)	3	(15.8)
Difficulty thinking clearly	2	(13.3)	0	(0.0)	2	(10.5)
Tingling WITH numbness	1	(6.7)	1	(25.0)	2	(10.5)
Difficulty concentrating	2	(13.3)	0	(0.0)	2	(10.5)
Tingling unspecified	1	(6.7)	1	(25.0)	2	(10.5)
All symptoms (physical & cognitive)	2	(13.3)	0	(0.0)	2	(10.5)
Trouble remembering	0	(0.0)	1	(25.0)	1	(5.3)
Tingling WITHOUT numbness	0	(0.0)	1	(25.0)	1	(5.3)
Muscle spasms	1	(6.7)	0	(0.0)	1	(5.3)
All cognitive symptoms	1	(6.7)	0	(0.0)	1	(5.3)
All physical symptoms	1	(6.7)	0	(0.0)	1	(5.3)
Difficulty finding the right words	1	(6.7)	0	(0.0)	1	(5.3)
Being sensitive to heat	1	(6.7)	0	(0.0)	1	(5.3)
Muscle weakness	1	(6.7)	0	(0.0)	1	(5.3)
Gastrointestinal symptoms	1	(6.7)	0	(0.0)	1	(5.3)

*Note* Percentages do not add to 100 as some participants reported multiple signs/symptoms as most bothersome

#### Hypoparathyroidism signs/symptoms viewed as most important to treat

Participants indicated a wide range of hypoparathyroidism physical and cognitive signs/symptoms that they considered most important to treat, and most participants mentioned multiple signs/symptoms (Table 3). The signs/symptoms that participants most often identified as most important to treat were feeling tired (31.6%, *n* = 6), muscle cramping (26.3%, *n* = 5), trouble remembering (26.3%, *n* = 5), pain (15.8%, *n* = 3), tingling with numbness (15.8%, *n* = 3), difficulty concentrating (15.8%, *n* = 3), difficulty finding the right words (15.8%, *n* = 3), low energy (15.8%, *n* = 3), and trouble sleeping (15.8%, *n* = 3):

…I think that one cannot work if you cannot cognitively function, so I think it’s important that people feel that they can focus, that they can read with comprehension, that they can participate in life and not feel so overwhelmed…The second part of it, I would say, is just to not have physical symptoms…I’ve really just had the physical pain. But I think you can’t live a full life if you’re in constant psychological and physical pain. (58-year-old female)

Three participants (15.8%) discussed all physical and cognitive signs/symptoms as most important to treat, and 3 participants (15.8%) mentioned all cognitive signs/symptoms as most important to treat.

**Table 3:** Hypoparathyroidism signs/symptoms participants viewed as most important to treat

*n* (%) reporting	TransCon PTH (*n* = 15)		Placebo (*n* = 4)		Total (*n* = 19)	
Feeling tired	5	(33.3)	1	(25.0)	6	(31.6)
Muscle cramping	4	(26.7)	1	(25.0)	5	(26.3)
Trouble remembering	4	(26.7)	1	(25.0)	5	(26.3)
Pain	2	(13.3)	1	(25.0)	3	(15.8)
Tingling WITH numbness	2	(13.3)	1	(25.0)	3	(15.8)
Difficulty concentrating	3	(20.0)	0	(0.0)	3	(15.8)
Difficulty finding the right words	3	(20.0)	0	(0.0)	3	(15.8)
Low energy	2	(13.3)	1	(25.0)	3	(15.8)
Trouble sleeping	2	(13.3)	1	(25.0)	3	(15.8)
All symptoms (physical & cognitive)	2	(13.3)	1	(25.0)	3	(15.8)
All cognitive symptoms	3	(20.0)	0	(0.0)	3	(15.8)
Muscle spasms	2	(13.3)	0	(0.0)	2	(10.5)
Difficulty understanding information	2	(13.3)	0	(0.0)	2	(10.5)
Muscle twitching	2	(13.3)	0	(0.0)	2	(10.5)
Tingling WITHOUT numbness	0	(0.0)	1	(25.0)	1	(5.3)
Muscle weakness	0	(0.0)	1	(25.0)	1	(5.3)
Heart problems	1	(6.7)	0	(0.0)	1	(5.3)
Trouble thinking clearly	1	(6.7)	0	(0.0)	1	(5.3)
Being sensitive to heat	1	(6.7)	0	(0.0)	1	(5.3)
Seizures	1	(6.7)	0	(0.0)	1	(5.3)
Unsure	1	(6.7)	0	(0.0)	1	(5.3)

*Note* Percentages do not add to 100 as some participants reported multiple signs/symptoms as most important to treat

### Treatment impacts on physical signs/symptoms

#### Trial treatment impacts on physical signs/symptoms

Participant reports of physical signs/symptoms experienced in the pre- and post-trial periods and the signs/symptoms that improved with the trial treatment are shown by trial treatment group in Table 4. The most frequently reported physical signs/symptoms in the period before starting the trial treatment were muscle twitching (94.7%, *n* = 18), low energy (94.7%, *n* = 18), feeling tired (89.5%, *n* = 17), muscle weakness (89.5%, *n* = 17), tingling without numbness (89.5%, *n* = 17), trouble sleeping (89.5%, *n* = 17), muscle cramping (84.2%, *n* = 16), tingling with numbness (84.2%, *n* = 16), muscle spasms (73.7%, *n* = 14), pain (73.7%, *n* = 14), heart problems (63.2%, *n* = 12), and sensitivity to heat (47.4%, *n* = 9).

For each of the most frequently reported physical signs/symptoms in the pre-trial period, a majority of participants who experienced the sign/symptom before the trial indicated that it improved with the trial treatment. Reported sign/symptom improvement with treatment was substantially higher for participants in the TransCon PTH group (range 84.6–100.0%) compared to the placebo group (range 25.0–50.0%) for all of the most frequently reported signs/symptoms.

**Table 4:** Experience of physical signs/symptoms pre- and post-trial and improvement with treatment

*n* (%) reporting	TransCon PTH (*n* = 15)						Placebo (*n* = 4)						Total (*n* = 19)					
	Pre-trial		Post-trial		Improved with Tx^a^		Pre-trial		Post-trial		Improved with Tx^a^		Pre-trial		Post-trial		Improved with Tx^a^	
Muscle twitching	15	(100.0)	4	(26.7)	15	(100.0)	3	(75.0)	1	(25.0)	1	(33.3)	18	(94.7)	5	(26.3)	16	(88.9)
Low energy	14	(93.3)	3	(20.0)	13	(92.9)	4	(100.0)	1	(25.0)	1	(25.0)	18	(94.7)	4	(21.1)	14	(77.8)
Feeling tired	13	(86.7)	5	(33.3)	12	(92.3)	4	(100.0)	1	(25.0)	1	(25.0)	17	(89.5)	6	(31.6)	13	(76.5)
Muscle weakness	14	(93.3)	1	(6.7)	13	(92.9)	3	(75.0)	1	(25.0)	1	(33.3)	17	(89.5)	2	(10.5)	14	(82.4)
Tingling WITHOUT numbness	13	(86.7)	4	(26.7)	11	(84.6)	4	(100.0)	3	(75.0)	1	(25.0)	17	(89.5)	7	(36.8)	12	(70.6)
Trouble sleeping	13	(86.7)	2	(13.3)	12	(92.3)	4	(100.0)	1	(25.0)	1	(25.0)	17	(89.5)	3	(15.8)	13	(76.5)
Muscle cramping	13	(86.7)	6	(40.0)	12	(92.3)	3	(75.0)	2	(50.0)	1	(33.3)	16	(84.2)	8	(42.1)	13	(81.3)
Tingling WITH numbness	13	(86.7)	6	(40.0)	12	(92.3)	3	(75.0)	2	(50.0)	1	(33.3)	16	(84.2)	8	(42.1)	13	(81.3)
Muscle spasms	12	(80.0)	1	(6.7)	12	(100.0)	2	(50.0)	0	(0.0)	1	(50.0)	14	(73.7)	1	(5.3)	13	(92.9)
Pain	11	(73.3)	3	(20.0)	10	(90.9)	3	(75.0)	1	(25.0)	1	(33.3)	14	(73.7)	4	(21.1)	11	(78.6)
Heart problems	9	(60.0)	4	(26.7)	6	(66.7)	3	(75.0)	2	(50.0)	1	(33.3)	12	(63.2)	6	(31.6)	7	(58.3)
Sensitivity to heat	7	(46.7)	1	(6.7)	6	(85.7)	2	(50.0)	1	(25.0)	1	(50.0)	9	(47.4)	2	(10.5)	7	(77.8)
Muscle tightness/stiffness	1	(6.7)	2	(13.3)	1	(100.0)	1	(25.0)	0	(0.0)	0	(0.0)	2	(10.5)	2	(10.5)	1	(50.0)
GI symptoms	1	(6.7)	0	(0.0)	0	(0.0)	0	(0.0)	0	(0.0)	–	–	1	(5.3)	0	(0.0)	0	(0.0)
Seizures	1	(6.7)	0	(0.0)	1	(100.0)	0	(0.0)	0	(0.0)	–	–	1	(5.3)	0	(0.0)	1	(100.0)
Physical vibration sensation	1	(6.7)	0	(0.0)	1	(100.0)	0	(0.0)	0	(0.0)	–	–	1	(5.3)	0	(0.0)	1	(100.0)
Slurred speech	1	(6.7)	0	(0.0)	1	(100.0)	0	(0.0)	0	(0.0)	–	–	1	(5.3)	0	(0.0)	1	(100.0)
Sensitivity to cold	1	(6.7)	0	(0.0)	0	(0.0)	0	(0.0)	0	(0.0)	–	–	1	(5.3)	0	(0.0)	0	(0.0)

*GI* gastrointestinal, *Tx* treatment

^a^Percent improved with treatment based on *n* of participants reporting sign/symptom pre-trial

#### Physical signs/symptoms most improved with treatment

**Fig. 1:**
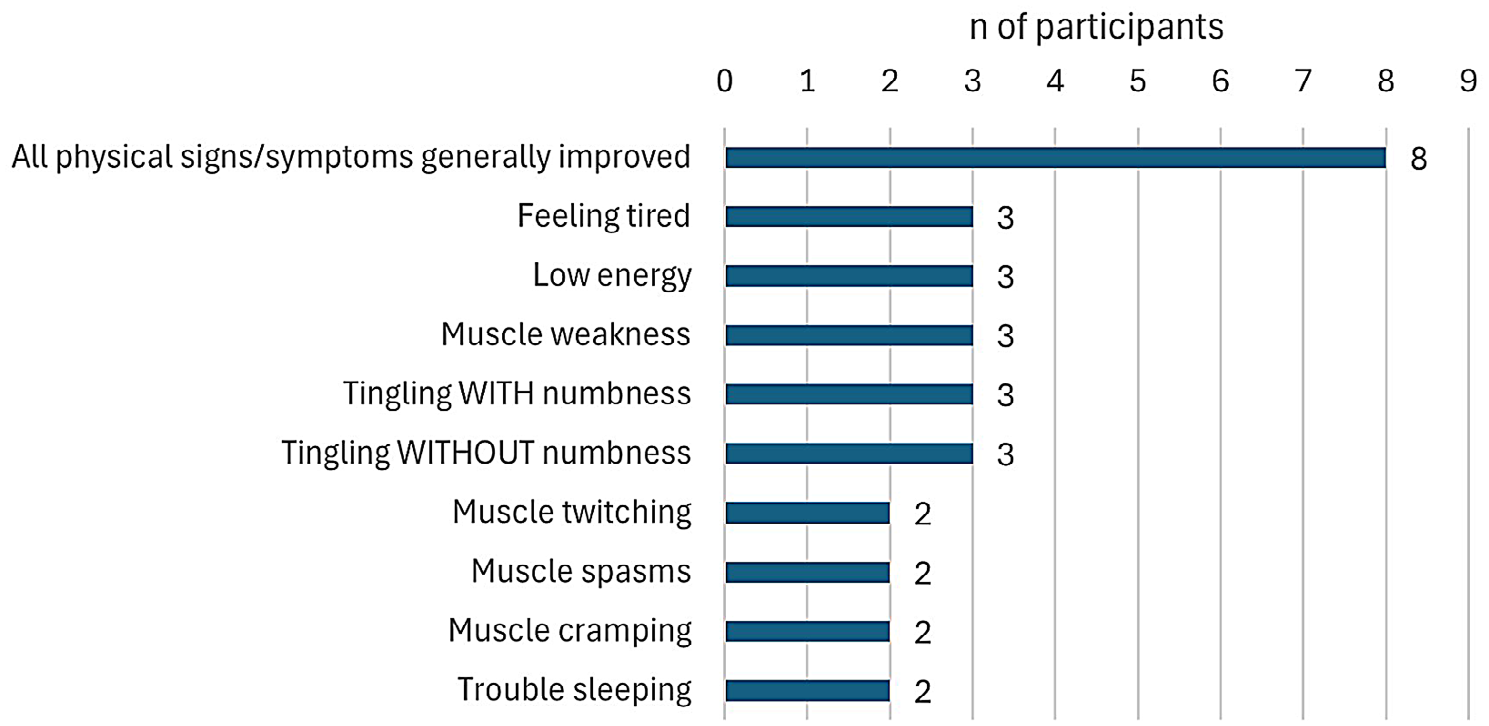
Physical signs/symptoms most improved with treatment for TransCon PTH group. *Note* Responses not mutually exclusive, *n* = 15

Participants who experienced any improvement in hypoparathyroidism physical symptoms over the trial period were asked whether some symptoms improved with treatment more than others. Among participants treated with TransCon PTH (*n* = 15), the physical signs/symptoms noted as most improved with treatment are shown in Fig. 1. More than half of participants receiving TransCon PTH (53.3%, *n* = 8) indicated that all of their physical signs and symptoms were generally improved over the course of the trial. The individual physical symptoms specified as most improved were among those participants reported as most bothersome and most important to treat.

Among those in the placebo group, only one participant (25.0%) reported improvement in physical symptoms; this participant indicated that, with the exception of tingling, all physical symptoms were generally improved.

### Treatment impacts on cognitive symptoms

#### Trial treatment impacts on cognitive symptoms

Participant reports of cognitive signs/symptoms that they experienced pre-trial and after their last blinded trial visit, as well as symptoms that improved with treatment, are shown by treatment group in Table 5. Among the total sample, the most commonly reported cognitive signs/symptoms prior to the trial period were difficulty finding the right words (89.5%, *n* = 17), difficulty concentrating (89.5%, *n* = 17), trouble remembering (84.2%, *n* = 16), trouble thinking clearly (84.2%, *n* = 16), and difficulty understanding information (68.4%, *n* = 13).

Rates of reported cognitive signs/symptoms were substantially lower in the post-trial period. A majority of participants observed that their cognitive signs/symptoms improved with treatment. Compared to the placebo group, participants in the TransCon PTH group were much more likely to report improvement of cognitive signs/symptoms with the trial treatment. For example, 14 participants (93.3%) in the TransCon PTH group reported improvement in difficulty concentrating compared to 1 participant (50.0%) in the placebo group.

**Table 5:** Experience of cognitive signs/symptoms pre- and post-trial and improvement with treatment

*n* (%) reporting	TransCon PTH (*n* = 15)						Placebo (*n* = 4)						Total (*n* = 19)					
	Pre-trial		Post-trial		Improved with Tx^a^		Pre-trial		Post-trial		Improved with Tx^a^		Pre-trial		Post-trial		Improved with Tx^a^	
Difficulty finding the right words	15	(100.0)	6	(40.0)	13	(86.7)	2	(50.0)	1	(25.0)	1	(50.0)	17	(89.5)	7	(36.8)	14	(82.4)
Difficulty concentrating	15	(100.0)	6	(40.0)	14	(93.3)	2	(50.0)	1	(25.0)	1	(50.0)	17	(89.5)	7	(36.8)	15	(88.2)
Trouble remembering	14	(93.3)	7	(46.7)	13	(92.9)	2	(50.0)	1	(25.0)	1	(50.0)	16	(84.2)	8	(42.1)	14	(87.5)
Trouble thinking clearly	14	(93.3)	3	(20.0)	12	(85.7)	2	(50.0)	1	(25.0)	1	(50.0)	16	(84.2)	4	(21.1)	13	(81.3)
Difficulty understanding information	12	(80.0)	3	(20.0)	10	(83.3)	1	(25.0)	0	(0.0)	1	(100.0)	13	(68.4)	3	(15.8)	11	(84.6)
Confusion	2	(13.3)	0	(0.0)	2	(100.0)	0	(0.0)	0	(0.0)	–	–	2	(10.5)	0	(0.0)	2	(100.0)

*Tx* treatment

^a^Percent improved with treatment based on *n* of participants who reported experiencing cognitive sign/symptom pre-trial

#### Cognitive signs/symptoms most improved with treatment

**Fig. 2:**
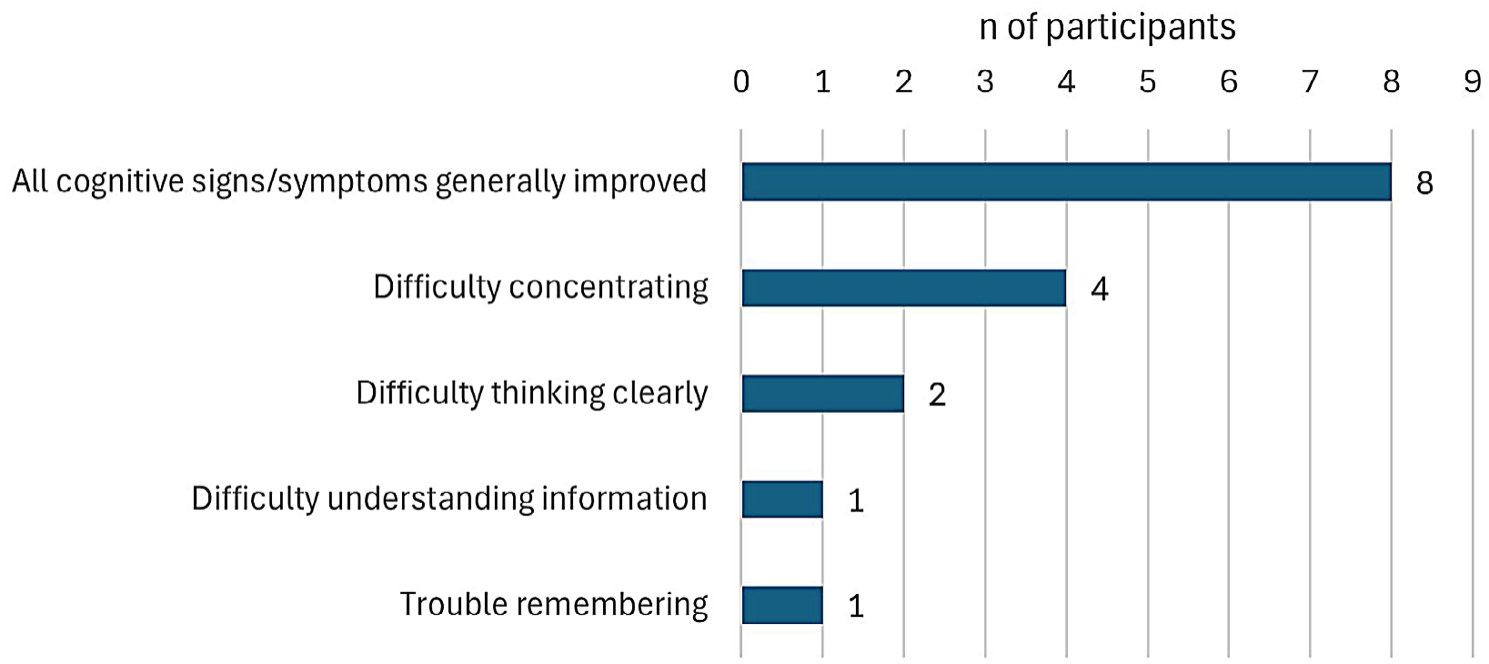
Cognitive signs/symptoms most improved with treatment in TransCon PTH group. *Note* Responses not mutually exclusive, *n* = 12

Participants who experienced any improvement in hypoparathyroidism cognitive signs/symptoms over the trial period were asked about whether some cognitive symptoms improved more than others. The cognitive symptoms reported as most improved with treatment among those responding in the TransCon PTH group (*n* = 12) are presented in Fig. 2. A majority of participants (66.7%, *n* = 8) indicated that all cognitive signs/symptoms had generally improved with treatment. Specific cognitive symptoms that were most often identified as the most improved with treatment were among those mentioned as the most bothersome symptoms and/or the symptoms most important to treat.

One participant in the placebo group described improvement in cognitive symptoms with the trial treatment but did not identify any cognitive symptoms as most improved.

### Understanding meaningful change in hypoparathyroidism symptoms

#### Meaningful change in overall hypoparathyroidism symptoms

To assess meaningful changes in overall hypoparathyroidism symptoms over the course of the blinded trial period, including both physical and cognitive symptoms, PGIS and PGIC questions were asked of participants during the interviews. Interviewees were then probed on the meaningfulness of any reported changes in overall symptoms from prior to starting trial treatment to the past 2 weeks/current time, as well as what other hypothetical changes in overall hypoparathyroidism symptoms would mean to them.

##### Overall symptom frequency—PGIS

Among those in the TransCon PTH group, 14 participants (93.3%) reported a reduced frequency of their overall hypoparathyroidism symptoms from the pre-trial period to the past 2 weeks on the PGIS. Reported differences in overall hypoparathyroidism symptom frequency ranged from improvements of 2-points to 5-points on the 6-point response option scale (ranging from “always” to “never”). All 14 participants who experienced an improvement in overall symptom frequency (100%) indicated that the change in the frequency of their overall hypoparathyroidism symptoms was meaningful to them (Table 6):

Yes, yes, it does…With physical fitness…or just my work…my work is physical because I’m always on the go…if I have a shift where I’m maybe doing more things that are a little bit more physical and it affects my ability to do those things, then it means more to me not to have that or to be able to exercise and not have symptoms and those things. Yes, it means a lot to me. (41-year-old female; response changed from “often” experience overall symptoms to “never” experience overall symptoms)

Three participants (20.0%) in the TransCon PTH group reported a 2-point improvement in overall symptom frequency on the response scale, and all reported that the change was meaningful. When asked if a 1-point lesser improvement on the response scale (a 1-point change) would still be meaningful to them, all 3 respondents (100%) indicated that it would be.

**Table 6:** Meaningful change in overall symptom frequency—PGIS

*n* (%) reporting^a^	TransCon PTH (*n* = 15)	Placebo (*n* = 4)
1 point improvement^b^	0	0
Improvement meaningful, yes^c^	–	–
2 points improvement	3 (20.0)	1 (25.0)
Improvement meaningful, yes	3 (100.0)	1 (100.0)
1 point less improvement would still be meaningful, yes	3 (100.0)	1 (100.0)
3 points or more improvement	11 (73.3)	0
Improvement meaningful, yes	11 (100.0)	–
1 point less improvement would still be meaningful, yes	11 (100.0)	–
Did not report Improvement	1 (6.7)	3 (75.0)

^a^Question and response options were based on the Patient Global Impression of Severity (PGIS). Response options were based on a 6-point scale and included never, occasionally, sometimes, often, very often, and always

^b^Reported improvements were based on the change in “points” on the response option scale from more frequent to less frequent over the trial period. A change from “often” to “sometimes” would be considered a 1-point improvement in symptom frequency; a change from “often” to “occasionally” would be considered a 2-point improvement in symptom frequency, etc

^c^Meaningful improvement was reported separately by level of improvement; subsample sizes vary

Eleven respondents (73.3%) in the TransCon PTH group noted a 3-point or greater improvement in overall symptom frequency on the response scale, and all reported that the change was meaningful. When asked if a 1-point lesser improvement would still make a meaningful difference to them, all 11 respondents (100%) affirmed that it would be meaningful.

Among those in the placebo group (*n* = 4), 1 participant (25.0%) reported less frequent overall symptoms from the pre-trial period to the past 2 weeks on the PGIS. This participant noted a 2-point improvement on the response scale and indicated that the change was meaningful.

##### Change in overall symptom frequency—PGIC

Among those receiving TransCon PTH (*n* = 15), 11 participants (73.3%) indicated experiencing overall symptoms much less often, 3 participants (20.0%) reported experiencing overall symptoms a little less often, and 1 participant (6.7%) reported no change in the frequency of overall symptoms (Table 7). For the 14 participants treated with TransCon PTH who experienced overall symptoms a little less or much less often following the blinded trial period compared to before starting the trial, all (100%) reported that the change made a meaningful difference to them:

…when I started to study, this disease was essentially my life. It was something that I always had to be managing and bringing with me everywhere and explaining to people and thinking about…I’m much more confident in the fact that I would be less symptomatic because of the medications, so I think the fact that I experience symptoms much less often gives me more freedom to feel more comfortable doing more things with more people and just having more natural experiences for my age. (27-year-old female; experienced overall symptoms “much less often” currently compared to before starting the trial)

**Table 7:** Meaningful change in overall symptom frequency—PGIC

*n* (%) reporting, change in frequency of overall symptoms^a^	TransCon PTH (*n* = 15)	Placebo (*n* = 4)
Much less often	11 (73.3)	1 (25.0)
Change meaningful, yes^b^	11 (100.0)	1 (100.0)
A little less often	3 (20.0)	1 (25.0)
Change meaningful, yes	3 (100.0)	1 (100.0)
No change	1 (6.7)	2 (50.0)
A little more often	0	0
Much more often	0	0

^a^Question and response options based on the PGIC (Patient Global Impression of Change)

^b^Meaningful change is reported separately for each level of change; subsample sizes vary

Among those in the placebo group (*n* = 4), 2 participants (50.0%) reported no change in overall symptoms over the course of the trial, 1 participant (25.0%) reported experiencing overall symptoms a little less often, and 1 participant (25.0%) indicated experiencing overall symptoms much less often. Both participants who experienced overall symptoms less frequently described the change as meaningful.

#### Meaningful change in physical symptoms

##### Physical symptom frequency—HPES

Among those receiving TransCon PTH, all 15 participants (100%) indicated a reduced frequency of physical symptoms from before starting trial treatment to the past 2 weeks on the HPES measure. Improvements in physical symptom frequency ranged from 1 to 4 points on the 5-point response scale (Table 8). All 15 participants (100%) indicated that the improvement in physical symptom frequency was meaningful to them:

I mentioned before my work, my day-to-day living, cooking, physical exercise…I feel better while I’m doing those things because I don’t have any symptoms. (41-year-old female; response changed from “often” experience physical symptoms to “never” experience physical symptoms)

**Table 8:** Meaningful change in physical symptom frequency—HPES

*n* (%) reporting^a^	TransCon PTH (*n* = 15)	Placebo (*n* = 4)
1 point improvement^b^	2 (13.3)	0
Improvement meaningful, yes^c^	2 (100.0)	–
2 points improvement	6 (40.0)	1 (25.0)
Improvement meaningful, yes	6 (100.0)	1 (100.0)
1 point less improvement would still be meaningful, yes	6 (100.0)	1 (100.0)
3 points or more improvement	7 (46.7)	0
Improvement meaningful, yes	7 (100.0)	–
1 point less improvement would still be meaningful, yes	7 (100.0)	–
Did not report improvement	0	3 (75.0)

^a^Question and response options were based on the Hypoparathyroidism Patient Experience Scale (HPES). Response options were based on a 5-point scale and included never, occasionally, sometimes, often, and very often/always

^b^Reported improvements were based on the change in “points” on the response option scale from more frequent to less frequent over the trial period. A change from “often” to “sometimes” would be considered a 1-point improvement in symptom frequency; a change from “often” to “occasionally” would be considered a 2-point improvement in symptom frequency, etc

^c^Meaningful improvement was reported separately by level of improvement; subsample sizes vary

##### Physical symptom frequency—PGIS

Among those receiving TransCon PTH, all 15 participants (100%) indicated a reduced frequency of physical symptoms from the time before starting the trial treatment to the past 2 weeks. Improvements in the frequency of physical symptoms ranged from 1 to 5 points on the 6-point response option scale, which ranged from “always” to “never” (Table 9). All 15 participants (100%) described the level of improvement they experienced in symptom frequency as meaningful to them.

**Table 9:** Meaningful change in physical symptom frequency—PGIS

*n* (%) reporting^a^	TransCon PTH (*n* = 15)	Placebo (*n* = 4)
1 point improvement^b^	3 (20.0)	0
Improvement meaningful, yes^c^	3 (100.0)	–
2 points improvement	4 (26.7)	1 (25.0)
Improvement meaningful, yes	4 (100.0)	1 (100.0)
1 point less improvement would still be meaningful, yes	4 (100.0)	1 (100.0)
3 points or more improvement	8 (53.3)	0
Improvement meaningful, yes	8 (100.0)	–
1 point less improvement would still be meaningful, yes	8 (100.0)	–
Did not report improvement	0	3 (75.0)

^a^Question and response options were based on the Patient Global Impression of Severity (PGIS). Response options were based on a 6-point scale and included never, occasionally, sometimes, often, very often, and always

^b^Reported improvements were based on the change in “points” on the response option scale from more frequent to less frequent over the trial period. A change from “often” to “sometimes” would be considered a 1-point improvement in symptom frequency; a change from “often” to “occasionally” would be considered a 2-point improvement in symptom frequency, etc

^c^Meaningful improvement was reported separately by level of improvement; subsample sizes vary

Among participants who received the placebo (*n* = 4), 1 respondent (25.0%) indicated a 2-point improvement in physical symptom frequency from before starting the trial treatment to the past 2 weeks, and this participant described the change as meaningful.

##### Change in physical symptom frequency—PGIC

Among those in the TransCon PTH treatment group (*n* = 15), 11 participants (73.3%) reported experiencing physical symptoms much less often, and 4 participants (26.7%) indicated a little less often (Table 10). All 15 participants (100%) in the TransCon PTH group confirmed that reduced frequency of their hypoparathyroidism physical symptoms made a difference that mattered to how they feel or function. Of those who received the placebo treatment, 1 participant (25.0%) reported experiencing physical symptoms much less often, and 3 participants (75.0%) reported no change from before starting the trial treatment to the current time.

**Table 10:** Meaningful change in physical symptom frequency—PGIC

*n* (%) reporting, change in frequency of physical symptoms^a^	TransCon PTH (*n* = 15)	Placebo (*n* = 4)
Much less often	11 (73.3)	1 (25.0)
Change meaningful, yes^b^	11 (100.0)	1 (100.0)
A little less often	4 (26.7)	0
Change meaningful, yes	4 (100.0)	–
No change	0	3 (75.0)
A little more often	0	0
Much more often	0	0

^a^Question and response options based on the PGIC (Patient Global Impression of Change)

^b^Meaningful change is reported separately for each level of change; subsample sizes vary

#### Meaningful change in cognitive symptoms

##### Cognitive symptom frequency—HPES

Of the 15 participants in the TransCon PTH group, 13 participants (86.7%) indicated improved cognitive symptom frequency from the time before starting trial treatment to the end of the blinded trial period. The changes in cognitive symptom frequency among respondents ranged from 1 point to 4 points on the response option scale (Table 11). Among those reporting reduced cognitive symptom frequency, all 13 participants (100%) noted that the change in cognitive symptom frequency made a difference that mattered to them in how they feel or function:

Yes, it does. Gives you a bit more confidence in yourself when you can remember a price that you were given five minutes ago and have to reiterate it five minutes later, that gives you confidence. (41-year-old female; response changed from “very often/always” experience cognitive symptoms to “often” experience cognitive symptoms)

**Table 11:** Meaningful change in cognitive symptom frequency—HPES

*n* (%) reporting^a^	TransCon PTH (*n* = 15)	Placebo (*n* = 4)
1 point improvement^b^	3 (20.0)	1 (25)
Improvement meaningful, yes^c^	3 (100.0)	1 (100)
2 points improvement	4 (26.7)	0
Improvement meaningful, yes	4 (100.0)	–
1 point less improvement would still be meaningful, yes	3 (75.0)	–
3 points or more improvement	6 (40.0)	0
Improvement meaningful, yes	6 (100.0)	–
1 point less improvement would still be meaningful, yes	6 (100.0)	–
Did not report improvement	2 (13.3)	3 (75.0)

^a^Question and response options were based on the Hypoparathyroidism Patient Experience Scale (HPES). Response options were based on a 5-point scale and included never, occasionally, sometimes, often, and very often/always

^b^Reported improvements were based on the change in “points” on the response option scale from more frequent to less frequent over the trial period. A change from “often” to “sometimes” would be considered a 1-point improvement in symptom frequency; a change from “often” to “occasionally” would be considered a 2-point improvement in symptom frequency, etc

^c^Meaningful improvement was reported separately by level of improvement; subsample sizes vary

Among participants who received the placebo treatment (*n* = 4), 1 respondent (25.0%) noted a 1-point improvement in cognitive symptom frequency on the HPES measure. This participant indicated that the change made a meaningful difference.

##### Cognitive symptom frequency—PGIS

Of the 15 participants who received the TransCon PTH trial treatment, 13 participants (86.7%) noted improvement in cognitive symptom frequency from the time before they started trial treatment to the past 2 weeks. Improvement ranged from 1 point to 5 points on the response option scale (Table 12). Among those reporting improvement in cognitive symptom frequency, all 13 participants (100%) described the change in cognitive symptom frequency that they experienced as meaningful:

Yes…there were definite aspects regarding the cognitive symptoms that were more severe on my mental health than the physical symptoms, such as…the embarrassment of being so forgetful, and the anxiety that the cognitive dysfunction gave me about, basically, feeling like I was losing my mind and wondering…what kind of impacts the cognitive dysfunction would have on my career…It’s a massive relief knowing that…the brain fog I had was validated, because the medication directly contributed to the improvement in the symptom. (27-year-old female; response changed from “very often” experience cognitive symptoms to “occasionally” experience cognitive symptoms)

Yes…it affects my ability to work and live and to do what I do. (41-year-old female; response changed from “often” experience cognitive symptoms to “occasionally” experience cognitive symptoms)

**Table 12:** Meaningful change in cognitive symptom frequency—PGIS

*n* (%) reporting^a^	TransCon PTH (*n* = 15)	Placebo (*n* = 4)
1 point improvement^b^	2 (13.3)	1 (25.0)
Improvement meaningful, yes^c^	2 (100.0)	1 (100.0)
2 points improvement	4 (26.7)	0
Improvement meaningful, yes	4 (100.0)	–
1 point less improvement would still be meaningful, yes	4 (100.0)	–
3 points or more improvement	7 (46.7)	0
Improvement meaningful, yes	7 (100.0)	–
1 point less improvement would still be meaningful, yes	6 (85.7)	–
Did not report improvement	2 (13.3)	3 (75.0)

^a^Question and response options were based on the Patient Global Impression of Severity (PGIS). Response options were based on a 6-point scale and included never, occasionally, sometimes, often, very often, and always

^b^Reported improvements were based on the change in “points” on the response option scale from more frequent to less frequent over the trial period. A change from “often” to “sometimes” would be considered a 1-point improvement in symptom frequency; a change from “often” to “occasionally” would be considered a 2-point improvement in symptom frequency, etc

^c^Meaningful improvement was reported separately by level of improvement; subsample sizes vary

In the placebo group (*n* = 4), 1 respondent (25.0%) reported a 1-point improvement in cognitive symptom frequency, and this participant described the change as meaningful.

##### Change in cognitive symptom frequency—PGIC

Among participants in the TransCon PTH treatment group (*n* = 15), 9 respondents (60.0%) reported experiencing symptoms much less often, 4 participants (26.7%) reported a little less often, and 2 participants (13.3%) reported no change (Table 13). For those in the TransCon PTH group who reported having cognitive symptoms less frequently, all 13 respondents (100%) described the change as meaningful:

…as a patient when you got brain fog and you’re in an appointment, you’re not able to process what’s going on, you never feel like you can put the right words out there to get your feelings and symptoms conveyed accurately. It’s been empowering as a patient to have some of those abilities return… To think clearly…be able to walk away from an experience and remember or at least think clearly about it. (39-year-old female; cognitive symptoms experienced “a little less often” currently compared to the start of the study)

**Table 13:** Meaningful change in cognitive symptom frequency—PGIC

*n* (%) reporting, change in frequency of overall symptoms^a^	TransCon PTH (*n* = 15)	Placebo (*n* = 4)
Much less often	9 (60.0)	0
Change meaningful, yes^b^	9 (100.0)	–
A little less often	4 (26.7)	1 (25.0)
Change meaningful, yes	4 (100.0)	1 (100.0)
No change	2 (13.3)	3 (75.0)
A little more often	0	0
Much more often	0	0

^a^Question and response options based on the PGIC (Patient Global Impression of Change)

^b^Meaningful change is reported separately for each level of change; subsample sizes vary

Among those in the placebo group, 1 participant (25.0%) reported experiencing cognitive symptoms a little less often, while 3 participants (75.0%) indicated no change in cognitive symptom frequency. The participant who noted experiencing cognitive symptoms a little less often affirmed that this change was meaningful.

### Trial treatment experience and satisfaction

Reports of overall trial treatment experiences varied by assigned treatment group. Most participants who received TransCon PTH (*n* = 15) described an overall positive treatment experience (86.7%, *n* = 13), while 2 participants (13.3%) reported a mixed experience. Among those in the placebo group (*n* = 4), 2 participants (50%) indicated a negative overall treatment experience, and 2 participants (50%) reported a mixed experience.

Interviewees who reported a positive overall treatment experience frequently described improvements in their symptoms, physical/daily functioning, and/or QOL that they attributed to the trial treatment:

I was tired and I had headaches, and it took a while I guess to get used to [the trial medication]. Then June 20, I remember it clearly, because I felt normal that day and I went on a date with my husband. We went and played putt putt, like this hadn’t happened in years to just have a regular day. Since June 20, I’ve felt fine. I’ve started working out again and I’ll have energy and I feel like my personality is back and I’m back to doing hobbies. I feel like I have my life back. Fatigue is gone, all the symptoms went away. It’s amazing. Very happy to be part of this trial. (42-year-old female)

Several participants, who were later identified as being in the placebo group, expressed that they did not have an overall positive treatment experience due to the lack of symptom improvement from the trial treatment they received.

Participant satisfaction with the trial treatment also varied by treatment group. Among those who received TransCon PTH (*n* = 15), 12 participants (80.0%) were very satisfied, and 3 participants (20.0%) were somewhat satisfied with the trial treatment. Interviewees who were very satisfied with the trial treatment described substantial improvements in their hypoparathyroidism symptoms and/or impacts on their QOL:

Very satisfied…Because of the result that it made for my life, physically and morally. Like, I kind of feel like I have a future now…before, I felt like when I was eating all them [calcium supplement] pills, I felt I was headed for trouble. (53-year-old male)

Participants who were somewhat satisfied mentioned still having some issues with hypoparathyroidism symptoms, calcium levels, and/or finding the appropriate trial treatment dosage.

Among respondents who received the placebo (*n* = 4), none were very satisfied with the trial treatment, 1 (25.0%) was somewhat satisfied, 1 (25.0%) was neither satisfied nor dissatisfied, 1 (25.0%) reported being somewhat dissatisfied, and 1 (25.0%) was very dissatisfied with the trial treatment. Participants who expressed dissatisfaction with the trial treatment mentioned their disappointment in their lack of improved hypoparathyroidism symptoms and/or their belief that they were receiving the placebo rather than the trial drug.

All interviewees who received TransCon PTH (100.0%, *n* = 15) described the treatment as effective/very effective or somewhat effective for the management of their hypoparathyroidism symptoms:

Very effective….I have zero cognitive symptoms now, and like I said, in the last several months, I’ve only had two days out of several months that I’ve had any physical symptoms, so to me that’s a huge success. (51-year-old female)

I would say it was fairly effective…pretty much total resolution of my cognitive problems, that was fantastic. It was a pretty good resolution of my physical symptoms. It wasn’t a total resolution, which is what I was hoping… But it’s certainly been enough that I’m happy with it and happy with how it’s been compared to how I was before. (47-year-old female)

Among participants who received the placebo (*n* = 4), 3 (75.0%) reported that the trial treatment was not effective in managing their hypoparathyroidism symptoms, and 1 (25.0%) reported that the treatment was somewhat effective:

…there was no change. I didn’t feel a difference in my symptoms, physical or cognitive. I didn’t see a difference in my lab results, so nothing. There is no difference. (43-year-old male)

Participants also discussed the trial treatment’s convenience and ease of use in response to general questions about their trial treatment experiences and what they liked or disliked about the trial treatment (Table 14).

**Table 14:** Ease of use and convenience of trial treatment by treatment group

	TransCon PTH (*n* = 15)		Placebo (*n* = 4)		Total (*n* = 19)	
Ease of use/convenience of trial treatment, *n* (%)						
Easy to administer	13	(86.7)	2	(50.0)	15	(78.9)
Single injection/dose	11	(73.3)	1	(25.0)	12	(63.2)
Fewer medications to take	5	(33.3)	1	(25.0)	6	(31.6)
Quick to administer	3	(20.0)	0	(0.0)	3	(15.8)
Like pen/pen works well	1	(6.7)	1	(25.0)	2	(10.5)
Able to self-inject	2	(13.3)	0	(0.0)	2	(10.5)
Easy to take medication with you	2	(13.3)	0	(0.0)	2	(10.5)
Does not require refrigeration after opening	2	(13.3)	0	(0.0)	2	(10.5)
Like cooler/packaging	1	(6.7)	0	(0.0)	1	(5.3)
Easy to remember medication	1	(6.7)	0	(0.0)	1	(5.3)
Flexible dose timing	1	(6.7)	0	(0.0)	1	(5.3)
Inconveniences/issues with use of trial treatment, *n* (%)						
Dislike of injections/needles	6	(40.0)	0	(0.0)	6	(31.6)
Issues with pen	3	(20.0)	0	(0.0)	3	(15.8)
Need for refrigeration prior to opening	2	(13.3)	0	(0.0)	2	(10.5)
Need to bring pen with you when traveling/away	1	(6.7)	0	(0.0)	1	(5.3)
Needing to dispose of sharps	1	(6.7)	0	(0.0)	1	(5.3)

### Trial treatment vs. pre-trial treatment comparisons and preferences

#### Previous treatment vs. trial treatment preferences

In total, 16 participants (84.2%) preferred the trial treatment, 2 participants (10.5%) preferred their pre-trial hypoparathyroidism treatment, and 1 participant (5.3%) had no preference.

Treatment preferences differed sharply by treatment group. Among those who received TransCon PTH, all 15 respondents (100%) indicated that they preferred the trial treatment compared to their previous hypoparathyroidism treatment:

I prefer the trial treatment. Definitely. (45-year-old female)

The trial treatment. One hundred, two hundred percent. (43-year-old female)

The more frequently given reasons for preferring the trial treatment compared to their previous hypoparathyroidism treatment among those in the TransCon PTH group were treatment efficacy/symptom improvement (93.3%, *n* = 14) and frequency of administration/single daily dose (66.7%, *n* = 10):

The ease of [the trial treatment] versus taking 15, 20 pills a day, and having to have a calcium all over your life of, in the office, in your purse, in the car. You can never be without it. (53-year-old female)

The trial has really given me a better quality of life. It’s given me a better feeling about my future, a sense of well-being. Big improvement…I have less to keep track of as far as them pills…and then the Vitamin D, like alternating days, some days I would eat three, some days I would have to eat two pills…You kind of have to keep track of what day you’re on…This is a lot more simple. You take the needle in the morning, and then it looks after itself. (53-year-old male)

Other reasons participants in the TransCon PTH group gave for their preference of the trial treatment compared to their previous hypoparathyroidism treatment were experiencing fewer side effects (33.3% *n* = 5) and improved QOL (26.7%, *n* = 4):

Interviewer: Okay. What are the reasons for your treatment preference?

Interviewee: Well, I was taking copious amounts of pills [with my previous treatment] that weren’t managing my symptoms. So, it was just keeping me alive and the fear of the damage that it would do on my organs long term with it and not having any quality of life with my prior stuff. So yes, the trial has changed that…Not only my quality of life but my longevity…The trial treatment is working for me… (43-year-old female).

…I would also say that [my previous treatment] sometimes it gave me bone pain and sometimes it gave me headaches, and I never had any of that on this drug at all. No side effects at all. (47-year-old female)

Among participants who received the placebo (*n* = 4), 2 (50.0%) preferred their previous treatment, 1 (25.0%) indicated no preference, and 1 (25.0%) preferred the trial treatment because of better symptom management.

### Views on whether received TransCon PTH or placebo during blinded period

Sixteen of the 19 participants (84.2%) believed that they received the trial drug (TransCon PTH); all but one were in the TransCon PTH group. The most often mentioned reasons given for believing they received TransCon PTH included being able to stop or reduce other medications/supplements (68.8%, *n* = 11), symptom improvement (56.3%, *n* = 9), and lab test results/bloodwork levels (e.g., calcium) (37.5%, *n* = 6).

Three participants (15.8%) believed that they received the placebo, and all 3 were assigned to the placebo group. The reasons participants gave for believing that they received the placebo included lack of symptom improvement (100%, *n* = 3), no change in lab test results/bloodwork levels (e.g., calcium) (66.7%, *n* = 2), and no change in other medications/supplements taken to treat hypoparathyroidism (66.7%, *n* = 2).

### Most important treatment impacts

**Fig. 3:**
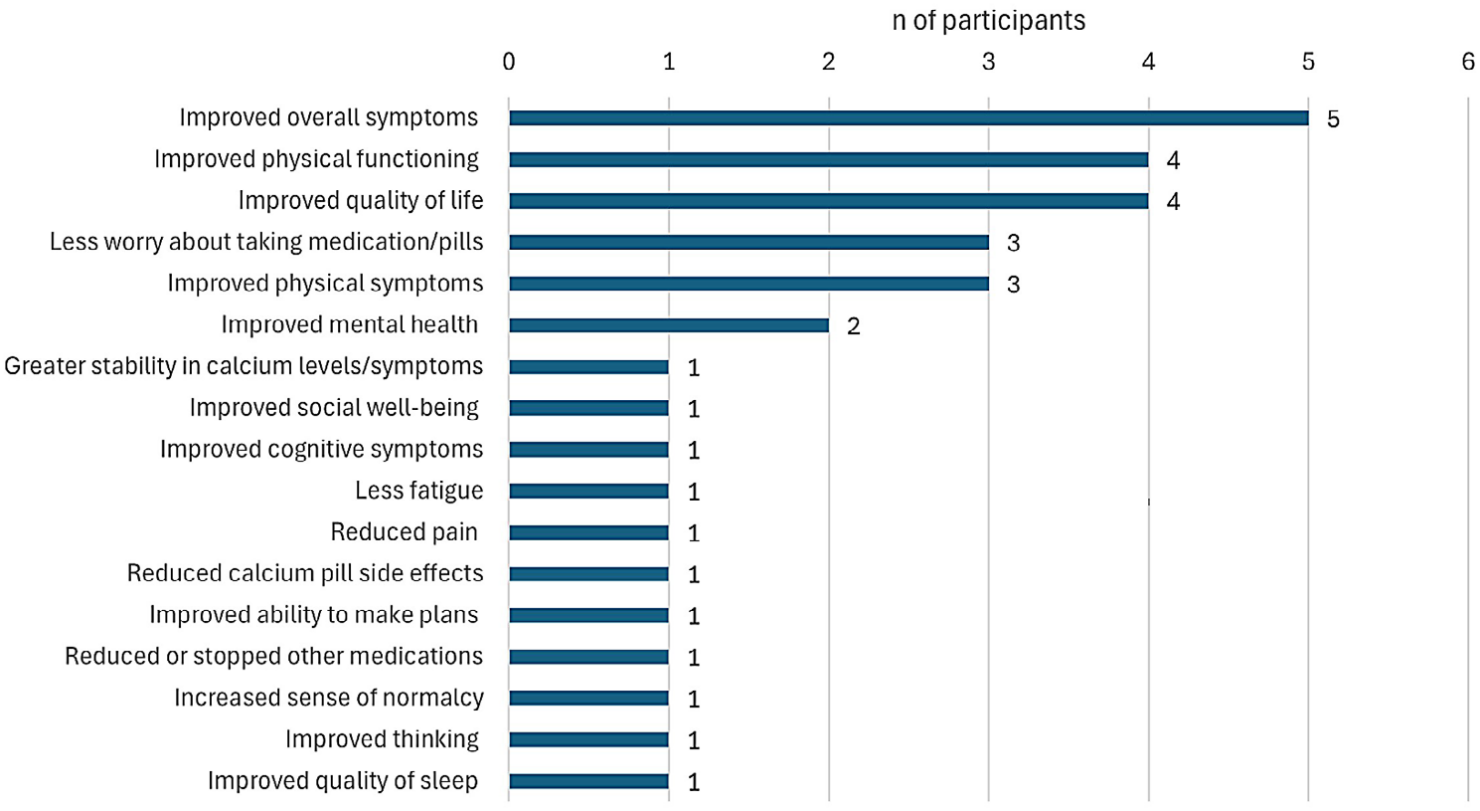
Most important treatment impacts overall for participants receiving TransCon PTH. *Note *Responses not mutually exclusive,* n* = 15

The most important overall treatment impacts reported by the 15 participants who received TransCon PTH are presented in Fig. 3:

Physically, I feel better. I mean, to me, that’s huge to not be in so much pain all the time…to feel better and not hurting all the time, that’s wonderful because it has been days when my husband’s like, oh, you want to go for a walk?…are you up for it? I’m like, oh yeah, let’s go for it. I feel good. Yeah, let’s go… (53-year-old female).

How I describe it to people is that I got my life back. I literally feel like I got my life back, and people around me can see that. People who know me well can see that. (58-year-old female)

Only 1 participant (25.0%) in the placebo group (*n* = 4) reported on the most important overall treatment impacts mentioning both improved overall symptoms and greater stability in calcium levels/symptoms as the most important impacts.

## Discussion

Participants in the TransCon PTH group reported marked improvement in overall, physical, and cognitive hypoparathyroidism symptoms, while those in the placebo group reported limited or no improvement. The vast majority of participants affirmed that the improvements they experienced in symptom frequency on the PGIS/PGIC and HPES–Symptom measures were meaningful, and that improvement occurred in those symptoms which were among those identified by participants as the most bothersome and most important to treat. Even small changes were repeatedly affirmed to make a difference that mattered to how participants feel or function. The study findings support the use of a 1-category improvement or change in the PGIS/PGIC and HPES–Symptom measures as being meaningful to adults with hypoparathyroidism.

It should be noted that the post-trial period referenced for the HPES measure questions, which refer to “the past 2 weeks,” varied among participants, depending on when they had their last trial visit (Visit 10). Participants were interviewed within 14 days of their last randomized trial visit, and the period from last trial visit to interview ranged from 0 to 14 days among interviewees. Thus, it is possible that some post-trial symptom improvements reported by those in the placebo group may have been due to their recent switch to TransCon PTH. One participant in the placebo group, for instance, reported that they experienced substantial symptom improvement with the new treatment they started following Visit 10. Further, information collected during the exit interview regarding change from the start of trial asked the respondent to think back to before they began the study, and this may have introduced some recall bias in their responses.

The interview findings have shown that trial participants who received TransCon PTH were generally satisfied with the treatment and found it to be effective or very effective for their hypoparathyroidism symptom management. Participants in the placebo group, in contrast, reported lower levels of satisfaction with the trial treatment and less efficacy for hypoparathyroidism symptom management. Many respondents appreciated that the trial treatment was easy to administer, required only a single daily injection/dose, and allowed them to take fewer medications or supplements. Respondents who received TransCon PTH unanimously preferred the trial treatment to their previous hypoparathyroidism treatment, primarily due to its efficacy/symptom improvement and the frequency of administration/single daily dose.

## Conclusions

The high degree of satisfaction with TransCon PTH and its reported efficacy for hypoparathyroidism symptom management among patients who received the drug during the phase 3 study suggest that TransCon PTH may be an important and effective new treatment option for patients with hypoparathyroidism. The findings indicate that TransCon PTH improved participants’ physical and cognitive hypoparathyroidism symptoms in meaningful ways, while reducing the daily treatment burden, e.g., fewer oral medications needed, associated with conventional therapy for hypoparathyroidism. In addition, the qualitative evidence from this study will be useful for future research aimed at understanding and interpreting meaningful changes in clinical and PRO symptom measures for adults with hypoparathyroidism.

## Data Availability

The data for the research presented in the publication may be available on a case-by-case basis for reasonable requests from the corresponding author.

## Abbreviations

HPES-Symptom: Hypoparathyroidism Patient Experience Scale-Symptom
IRB: Institution Review Board
PGIC: Patient Global Impression of Change
PGIS: Patient Global Impression of Severity
PRO: Patient-reported outcome
PTH: Parathyroid hormone
QOL: Quality of life
US: United States
